# MOBA eSports participation and psychological traits in individuals with disabilities—an association analysis grounded in self-efficacy, social support, and trait sport confidence

**DOI:** 10.3389/fpsyg.2026.1874127

**Published:** 2026-07-30

**Authors:** Yang Liu, Shengsheng Li, Hanxiang Lv, Kexiang Qian

**Affiliations:** 1 School of Physical Education, Hangzhou Normal University, Hangzhou, Zhejiang, China; 2 Zhejiang College of Construction, Hangzhou, Zhejiang, China

**Keywords:** eSports, individuals with disabilities, MOBA games, self-efficacy, social support, trait sport confidence

## Abstract

**Objective:**

This study examined β-efficacy, social support, and trait sport confidence among individuals with disabilities, and investigated the predictive roles of participation-related characteristics.

**Methods:**

Convenience sampling recruited 114 participants playing Honor of Kings. Psychological traits were assessed using GSES, ISEL-SF, and TSCI. Analyses included *t*-tests, ANOVA, hierarchical regression, and multiple regression.

**Results:**

(1) The eSports group scored significantly higher on all three psychological measures (*p* < 0.001; Cohen’s *d* = −1.17 to −2.19). (2) Daily participation duration showed a U-shaped relationship with self-efficacy and trait sport confidence (both *p* < 0.01). (3) eSports performance positively predicted self-efficacy (*β* = 0.418, *p* = 0.005); years of participation showed no significant effects.

**Conclusion:**

MOBA eSports participation is positively associated with psychological traits in disabled individuals, yet the U-shaped duration-effect relationship highlights the need for moderation. Competitive performance predicts self-efficacy, while social support relates primarily to offline relationships. eSports may empower this population psychologically, but risks and boundary conditions require further investigation.

## 1 Introduction

The equal participation of persons with disabilities in social, cultural, and sports activities, alongside the promotion of their mental health development, constitutes a fundamental goal of China’s disability affairs agenda and the construction of an inclusive society. The 14th Five-Year Plan for the Protection and Development of Persons with Disabilities explicitly calls for enriching the spiritual and cultural lives of disabled individuals. It also promotes the coordinated development of mass sports, rehabilitative sports, and competitive sports for this population, and encourages the expansion of social participation channels through digital technologies. The newly revised Sports Law of the People’s Republic of China (2022) guarantees the legitimate rights of disabled individuals to participate equally in various sports activities. It also encourages the development of novel sports formats adapted to this population, aiming to break down the participation barriers inherent in traditional sports (General Administration of Sport of China, 2022). However, current policy implementation in China has largely focused on surface-level practices such as event organization and infrastructural modifications. In-depth investigation into the effects of eSports participation on the internal mental health of disabled individuals remains insufficient.

From the perspective of mental health among disabled populations, physical functional limitations and reduced social participation opportunities render depression, anxiety, and post-traumatic stress disorder the most prevalent mental health challenges in this group (Sang-ho, 2022). Existing research indicates that persons with disabilities commonly exhibit psychological deficits, including insufficient self-efficacy, weak perceived social support, and low self-confidence. At the level of self-efficacy, physical impairments and diminished social engagement continuously undermine general self-efficacy. This leads individuals to develop negative appraisals of their capacity to cope with life challenges and accomplish daily tasks, making them more susceptible to withdrawal and avoidance behaviors when confronted with adversities (Asghari, 2001), which further reduces their willingness to actively participate in social activities. For instance, a qualitative study found that some men with spinal cord injuries avoided social interactions due to feeling inferior to their socially active peers (Tagaki, 2016). Regarding social support, existing research demonstrates that disabled individuals receive generally lower levels of social support and face structural barriers—including policy gaps, geographical disparities, service integration difficulties, cultural mismatches, and socioeconomic marginalization—in accessing social support services (Fortune et al., 2021). In terms of self-confidence, prolonged life adversities, external stereotypes, and social isolation persistently deplete trait-level self-confidence. Diminished self-confidence in turn impedes social adaptation, interpersonal engagement, and self-development, ultimately forming a vicious cycle of “physical limitations—social withdrawal—psychological negativity” (Wang, 2001).

In response to the aforementioned difficulties faced by disabled individuals, a substantial body of research has demonstrated the significant ameliorative effects of physical activity. Data from the World Health Organization indicate that disabled individuals who regularly participate in sports exhibit a 45% lower prevalence of depressive symptoms compared to their physically inactive counterparts, with exercise-induced endorphin secretion constituting a “natural antidepressant barrier” (World Health Organization, 2022). By executing specific motor actions, achieving training goals, or participating in competitions, disabled individuals accumulate experiential evidence of “I can do it,” thereby progressively reversing the negative self-appraisals formed through prolonged physical restrictions (Bandura, 1997). Physical exercise facilitates a role transition from “care recipient” to “athlete,” which helps attenuate the internalized effects of cumulative external stereotypes and enhances individuals’ sense of social identity (Hsu and Chiang, 2023). Furthermore, through cooperation and interaction within sports teams, disabled individuals may compensate for the structural deficiencies in social support services (Fortune et al., 2021). Although extensive research confirms the positive effects of physical exercise on the mental health of disabled individuals, these effects are not uniformly beneficial. A study of Paralympic athletes found that competitive pressure and performance expectations constituted the primary factors contributing to declines in athletic self-confidence (Mitić et al., 2021). Some athletes derive their confidence from uncontrollable external factors, rendering this “fragile confidence” highly susceptible to collapse in the face of setbacks (Vealey, 1986). This suggests that the psychological effects of physical exercise on disabled populations are twofold, and the psychological benefits of the same sport vary significantly across different disability groups or disciplines (Luca Puce et al., 2023).

As a sports category, eSports has been officially recognized as a formal sporting activity in countries including China, South Korea, and the United States, and has been incorporated into major international multi-sport events such as the Asian Games (Tang et al., 2025). Moreover, comparative studies between eSports players and traditional athletes have revealed no significant differences in anxiety levels, yet eSports players scored significantly higher on self-confidence dimensions, directly supporting a positive association between eSports participation and athletic self-confidence (Šunje, 2023). Drawing upon Social Cognitive Theory and self-efficacy theory as an analytical framework, this study posits that social support, as a critical external environmental resource, provides positive feedback for the establishment of individuals’ capability beliefs. Self-efficacy, as a core intrinsic psychological mechanism, is directly influenced by participation experiences and competitive performance, while also serving as a fundamental basis for the formation of trait athletic self-confidence. Furthermore, these three psychological traits interact synergistically to collectively reflect the comprehensive psychological state of disabled individuals following eSports participation. Accordingly, the following hypotheses are proposed:

*H1*: Disabled individuals who participate in eSports demonstrate significantly higher levels of self-efficacy, social support, and trait athletic self-confidence compared to their non-participating counterparts.

*H2*: eSports participation variables exert differential effects on the psychological traits of disabled individuals.

The frequency of participation in eSports competitions is positively correlated with social self-efficacy, whereas the frequency of participation in gaming communities is negatively correlated with social self-efficacy (Choi et al., 2026). Concurrently, domestic research has indicated that disabled players can achieve higher rankings by regulating their own behaviors. The longer the duration of eSports participation, the more proficiently players employ self-regulation strategies. Sustained deliberate practice gradually crystallizes into stable competitive competence, and the accumulation of competence directly and positively affects the enhancement of self-efficacy (Miao et al., 2024). From the perspective of self-efficacy theory, the improvement of competitive skills and the continuous receipt of positive in-game feedback constitute key sources for constructing positive capability beliefs and enhancing self-efficacy (Bandura, 1997). Accordingly, the following hypothesis is proposed:

*H2a*: Years of eSports participation and eSports performance (rank) significantly predict self-efficacy.

The relationship between daily eSports participation duration and psychological traits exhibits complex and multifaceted characteristics. Some scholars have suggested that moderate daily eSports participation can expand social networks through team-based competition and online interaction, continuously yielding a sense of competitive achievement and peer recognition, thereby steadily enhancing individual self-efficacy (Chung et al., 2020) and promoting positive affect and social connectedness (Shan et al., 2023). However, as daily participation duration increases, the observed patterns shift. Regarding self-efficacy, research indicates that prolonged immersion in MOBA games tends to impair users’ emotion regulation abilities (Luo, 2025). The same study noted that players with an average daily gaming time exceeding 6 h exhibited pronounced addictive tendencies, with gaming preoccupation adversely affecting work efficiency and family relationships. In terms of self-confidence, the study showed that players who remain immersed in MOBA games for extended periods are prone to extreme emotional reactions when experiencing in-game defeats (Luo, 2025). The present study proposes the following hypothesis in response to these patterns:

*H2b*: Average daily eSports participation duration exhibits a nonlinear relationship with self-efficacy, social support, and trait athletic self-confidence.

The relationship between years of gaming participation and social support is not direct but rather moderated by motivation and context, and entails certain potential risks (Luo et al., 2023). A study of college students in the United States and Italy indicated that excessive eSports involvement may be accompanied by risks including addiction, health impairment, and social isolation (Delello et al., 2023). For disabled individuals, who already face the practical predicament of limited social opportunities, the risk of over-reliance on the virtual world as a “refuge” may be particularly pronounced. Other research has shown that social support and self-regulatory capacity increase with players’ competitive rankings, suggesting that higher-level players possess stronger social support (Freeman and Wohn, 2017). Based on these findings, the following hypothesis is proposed:

*H2c*: Years of eSports participation and eSports performance significantly predict social support.

eSports also exert negative effects on mental health, particularly when participants lack sufficient self-control. Prolonged engagement in competitive gaming, exposure to pressure, and the pursuit of victory may subject players to excessive psychological stress, which can sometimes escalate into symptoms of anxiety and depression (Sun and Zhang, 2024). Furthermore, disabled individuals face additional challenges in eSports participation, including high costs of assistive technology adaptation and inadequate accessible design (Spöhrer and Ochsner, 2024), which may indirectly compromise competitive performance and paradoxically exacerbate feelings of frustration. The present study therefore proposes the following hypothesis:

*H2d*: Years of eSports participation and eSports performance (rank) significantly and positively predict trait athletic self-confidence.

Current research on eSports participation among disabled individuals exhibits notable limitations. The academic literature lacks systematic quantitative data on the scale, demographic characteristics, and participation patterns of this population (Hassan et al., 2025). Within media studies, cultural studies, game studies, and disability studies, self-identified disabled game players remain an “under-researched population” (Spöhrer and Ochsner, 2024). Direct empirical evidence comparing whether disabled eSports participants exhibit superior psychological outcomes—in terms of self-efficacy, social support, and trait athletic self-confidence—relative to non-participants remains scarce.

The present study focuses on the impact of eSports participation on the overall life adaptability of disabled individuals, while taking into account the special needs of this population in digital sports participation, including assistive technology adaptation and social integration. Drawing upon Bandura’s self-efficacy theory, the operational confidence cultivated within eSports contexts represents only situation-specific competence, which inadequately reflects the comprehensive psychological resources required for daily life, interpersonal interaction, and social integration. Therefore, this study adopts general self-efficacy as the core variable to measure transferable capability beliefs applicable across various life domains, and provides an empirical examination of eSports participation among disabled individuals in relation to their self-efficacy, social support, and trait athletic self-confidence.

## 2 Methods

### 2.1 Participants

This study employed a convenience sampling method to recruit participants with disabilities under the age of 60 through provincial-level disabled persons’ federations, disability sports associations, and related eSports communities between March and May 2024. Clear inclusion and exclusion criteria were established: (a) participants were required to hold a legal disability certification and be capable of independently understanding and completing the questionnaire; (b) individuals with cognitive impairments, impaired consciousness, or inability to independently cooperate with the survey were excluded. To control for the potential confounding effects of game type heterogeneity on the study results, all participants were uniformly required to engage in only one popular multiplayer online battle arena (MOBA) eSports title.

This study received prior approval from the Ethics Review Committee (approval number: 20240501) and strictly adhered to the ethical principles of the Declaration of Helsinki throughout the process. A total of 120 questionnaires were distributed; after excluding those with patterned responses, excessive missing data, or invalidity, 114 valid questionnaires were obtained, yielding an effective response rate of 95%. Based on a 6-month eSports participation history, the sample was divided into an eSports participation group and a non-participation group, with 57 participants in each. The demographic and disability-related characteristics of the sample are presented in Table 1.

**Table 1:** Baseline demographic and disability-related characteristics of participants in the eSports participation and non-participation groups.

Variable	Category	eSports participation group (*N* = 57)	Non-eSports control group (*N* = 57)	Total (*N* = 114)
Gender	Male	48 (84.2%)	28 (49.1%)	76
	Female	9 (15.8%)	29 (50.9%)	38
Age	<18 years	14 (24.6%)	7 (12.3%)	21
	18 years-25 years	29 (50.9%)	21 (36.8%)	50
	25 years-30 years	10 (17.5%)	13 (22.8%)	23
	>30 years	4 (7.0%)	16 (28.1%)	20
Marital status	Unmarried	49 (86%)	28 (49.1%)	77
	Married	3 (5.3%)	21 (36.8%)	24
	Divorced	0 (0%)	3 (5.3%)	3
	Widowed	5 (8.8%)	5 (8.8%)	10
Educational attainment	Primary school	2 (3.5%)	8 (14%)	10
	Junior high school	9 (15.8%)	21 (36.8%)	30
	High school or secondary technical school	21 (36.8%)	20 (35.1%)	41
	College degree or above	25 (43.9%)	8 (14%)	33
Monthly basic income	<2 K	24 (42.1%)	20 (35.1%)	44
	2 K-5 K	19 (33.3%)	23 (40.4%)	42
	5 K-8 K	7 (12.3%)	14 (24.5%)	21
	8 K-10 K	1 (1.8%)	0 (0%)	1
	>10 K	6 (10.5%)	0 (0%)	6
Disability type	Hearing disability	46 (80.7%)	13 (22.8%)	59
	Speech disability	2 (3.5%)	15 (26.3%)	17
	Physical disability	9 (15.8%)	29 (50.9%)	38
Disability grade	Grade 1	38 (66.7%)	10 (17.5%)	48
	Grade 2	9 (15.7%)	14 (24.6%)	23
	Grade 3	5 (8.8%)	24 (42.1%)	29
	Grade 4	5 (8.8%)	9 (15.8%)	14

### 2.2 Measurement instruments

#### 2.2.1 General self-efficacy scale (GSES)

This study employed the General Self-Efficacy Scale (GSES), originally developed by Schwarzer et al. and translated and revised by Wang et al. (2001), to measure individuals’ general self-efficacy in coping with various situations. The GSES was selected over eSports-specific self-efficacy instruments for the following reasons: (a) the study focuses on the impact of eSports participation on the overarching, cross-situational general self-efficacy of individuals with disabilities, rather than on game-context-specific efficacy, for which the GSES provides appropriate psychometric capture; (b) existing eSports-specific self-efficacy scales lack validated applicability among disability populations, whereas the Chinese version of the GSES has been extensively utilized and well-validated in this demographic. The scale consists of 10 items rated on a 4-point Likert scale ranging from 1 (“completely inconsistent”) to 4 (“completely consistent”), with higher total scores indicating higher levels of general self-efficacy. In this study, the Cronbach’s *α* coefficient for the GSES was 0.926, the KMO value was 0.928, and Bartlett’s test of sphericity yielded a significance level of *p* < 0.01, indicating satisfactory reliability and validity.

#### 2.2.2 Social support scale (ISEL-SF)

This study adopted the Interpersonal Support Evaluation List–Short Form (ISEL-SF), which comprises 16 items rated on a 4-point scale ranging from “strongly agree” to “strongly disagree” (Payne et al., 2012). The scale assesses four dimensions: (a) Appraisal Support (AP), reflecting perceived availability of someone to discuss personally important issues; (b) Tangible Assistance (TA), reflecting perceived availability of material aid; (c) Belonging Support (BE), reflecting perceived availability of others for social interaction; and (d) Self-Esteem Support (SE), reflecting perceived availability of a positive comparison with others. Certain items are reverse-scored and require raw-score transformation according to the formula: transformed score = 5 − original item score (i.e., “strongly agree” = 1, “strongly disagree” = 4), whereas the remaining items are positively scored. Higher total scores indicate higher levels of perceived social support. In this study, the Cronbach’s *α* coefficient for the ISEL-SF was 0.874, the KMO value was 0.895, and Bartlett’s test of sphericity yielded a significance level of *p* < 0.01, demonstrating good reliability and validity.

#### 2.2.3 Trait sport-confidence inventory (TSCI)

This study employed the Trait Sport-Confidence Inventory (TSCI), which consists of 8 items rated on a 4-point scale ranging from “strongly agree” to “completely disagree,” with all items positively scored (Shen, 2004). Higher scores indicate greater trait athletic self-confidence. In this study, the Cronbach’s α coefficient for the TSCI was 0.933, the KMO value was 0.895, and Bartlett’s test of sphericity yielded a significance level of *p* < 0.01, indicating satisfactory reliability and validity.

#### 2.2.4 Self-developed questionnaire on MOBA eSports basic information

The control variables in this study included gender, age, marital status, educational attainment, monthly basic income, disability type, and disability grade (Zhang and Wu, 2015; Zhang, 2014). The demographic and disability-related characteristics of the sample are presented in Table 1. MOBA eSports participation variables encompassed average daily eSports participation duration, years of eSports participation, and eSports performance (rank). Following the completion of the preliminary draft of the self-developed MOBA eSports participation questionnaire, a standardized expert content validity review was conducted. Five experts in sport psychology, mental health of individuals with disabilities, and eSports were invited to undertake two rounds of review: in the first round, experts evaluated the relevance of all items to the research topic and target population, leading to the elimination of items with weak relevance; in the second round, the questionnaire content was refined based on expert feedback regarding wording, expression, and contextual appropriateness. The Cronbach’s *α* coefficient for the self-developed MOBA eSports participation questionnaire was 0.648, the KMO value was 0.590, and Bartlett’s test of sphericity yielded a significance level of *p* < 0.01, indicating significant inter-item correlations that satisfy the prerequisite for factor analysis and confer basic psychometric reliability. eSports performance (rank) was categorized into four ordinal groups: Basic Rank Group, Intermediate Rank Group, Advanced Rank Group, and Elite Rank Group, representing a clear hierarchical order of competitive skill levels. In one-way analysis of variance (ANOVA), the original rank grouping was directly employed as the grouping factor for between-group mean comparisons. In multiple linear regression analysis, the four-level rank variable was transformed via ordinal assignment and entered into the model as an ordinal metric predictor. This approach was adopted in accordance with the sample characteristics of this study and prevailing practices in the field.

### 2.3 Statistical analysis

SPSS 27.0 was used for all statistical analyses. First, descriptive statistics were conducted to summarize demographic characteristics, disability-related information and scores of all psychological scales across the full sample. Independent samples *t*-tests were performed to compare the three psychological traits (self-efficacy, social support, trait sport confidence) between the MOBA eSports group and the non-eSports control group among all participants.

One-way ANOVA, hierarchical multiple linear regression and multiple linear regression analyses were only conducted within the MOBA eSports participation subgroup (*n* = 57). One-way ANOVA was adopted to examine differences in the three psychological indicators across subgroups with varying daily gaming duration, years of participation and competitive ranks. Hierarchical multiple linear regression was used to test the curvilinear predictive effects of daily eSports participation duration on the three psychological traits. Multiple linear regression was further applied to analyze the predictive effects of years of eSports participation and competitive rank, with disability type, gender, age, marital status, educational attainment and disability grade entered as control variables to identify their influences on core outcomes.

Prior to regression analyses, Shapiro–Wilk test for normality and Levene’s test for homogeneity of variances were implemented, and all results satisfied the prerequisites for parametric tests. Variance inflation factor (VIF < 3) was calculated to detect multicollinearity, eliminating collinearity bias and ensuring the robustness of statistical results.

## 3 Results

### 3.1 Baseline comparison of demographic variables

As shown in Table 2, the two groups showed no statistically significant difference only in monthly basic income. Significant between-group differences were found for the other six indicators—gender, age, marital status, educational attainment, disability type, and disability grade (*p* < 0.05)—indicating baseline imbalance. Accordingly, in the subsequent multiple linear regression analyses, the above six indicators with significant between-group differences were included as control variables in the regression model. This was done to adjust for potential confounding bias arising from baseline imbalance.

**Table 2:** Baseline demographic comparisons between the MOBA eSports participation group and non-participation group.

Variable	eSports group (*n* = 57) x ± s	Non-eSports group (*n* = 57) x ± s	*t*	*p*
Gender	1.16 ± 0.368	1.51 ± 0.504	−4.243	<0.001
Age	2.07 ± 0.842	2.67 ± 1.024	−3.398	<0.001
Marital status	1.32 ± 0.869	1.74 ± 0.917	−2.516	0.013
Educational attainment	3.21 ± 0.840	2.49 ± 0.909	4.389	<0.001
Monthly basic income	2.05 ± 1.260	1.89 ± 0.772	0.807	0.422
Disability type	1.35 ± 0.744	2.28 ± 0.818	−6.348	<0.001
Disability grade	1.60 ± 0.979	2.56 ± 0.964	−5.301	<0.001

Adjusted *t*-test was used when variances were unequal; *p* < 0.05 indicates a statistically significant between-group difference.

### 3.2 Comparison of psychological traits between disabled individuals participating in MOBA eSports and those not participating

In Table 3, in this study, independent samples *t*-tests were conducted on 57 participants in each group (general disabled group and MOBA eSports disabled group). The results showed that the MOBA eSports disabled group scored significantly higher than the general disabled group on social support, trait sport confidence, and self-efficacy (social support: *t* = −8.719, *p* < 0.001, Cohen’s *d* = −1.63; trait sport confidence: *t* = −11.678, *p* < 0.001, Cohen’s *d* = −2.19; self-efficacy: *t* = −6.240, *p* < 0.001, Cohen’s *d* = −1.17). All differences reached statistical significance with large effect sizes, indicating that disabled individuals participating in MOBA eSports activities demonstrated significant advantages in self-efficacy, trait sport confidence, and social support.

**Table 3:** Comparison of psychological traits between disabled individuals participating and not participating in esports (M ± SD, *n* = 114).

Variable	Disabled individuals participating in MOBA eSports (*n* = 57)	Disabled individuals not participating in eSports (*n* = 57)	*t*-value	Cohen’s *d*	*p*
Self-efficacy	26.86 ± 6.67	18.67 ± 7.33	−6.240	−1.17	≤0.001
Social support	46.33 ± 6.72	35.32 ± 6.77	−8.719	−1.63	≤0.001
Trait sport confidence	26.44 ± 4.26	17.63 ± 3.78	−11.678	−2.19	≤0.001

### 3.3 Differential effects of MOBA eSports participation factors on psychological traits of disabled individuals

One-way ANOVA was conducted to examine the differential effects of daily MOBA eSports participation duration, years of experience, and rank level on self-efficacy, social support, and trait sport confidence among disabled individuals, with results presented in Table 4. Daily participation duration had a significant effect on self-efficacy (*F* = 24.91, *p* < 0.001) and trait sport confidence (*F* = 4.178, *p* = 0.01), but no significant effect on social support. Years of experience showed no significant effects on any of the three psychological traits. Rank level significantly affected only self-efficacy (*F* = 3.793, *p* = 0.015), with no significant effects on social support or trait sport confidence.

**Table 4:** Differences in psychological traits of disabled individuals across MOBA eSports participation factors.

	*n*	Self-efficacy	Social support	Trait sport confidence
Daily participation duration				
<2 h	10	33.300 ± 4.832	35.100 ± 7.709	29.700 ± 3.945
2–4 h	16	24.875 ± 4.256	36.250 ± 6.962	25.312 ± 4.174
4–6 h	18	21.111 ± 3.909	36.167 ± 7.318	24.777 ± 3.750
>6 h	13	33.3 ± 4.939	33.154 ± 5.064	27.615 ± 3.885
*F*-value		24.91	0.628	4.178
*p*-value		< 0.001	0.600	0.01
Years of participation				
<3 years	19	25.105 ± 5.714	34.737 ± 5.258	27.421 ± 3.776
3–6 years	17	27.059 ± 7.521	36.824 ± 8.398	24.471 ± 2.853
>6 years	21	28.286 ± 6.702	34.619 ± 6.659	27.143 ± 5.170
*F*-value		1.150	0.593	2.772
*p*-value		0.324	0.556	0.071
Rank				
Basic rank group	10	24.500 ± 6.587	38.500 ± 8.182	25.000 ± 4.243
Intermediate rank group	11	24.091 ± 4.437	33.909 ± 5.839	24.182 ± 4.813
Advanced rank	13	25.077 ± 6.714	36.077 ± 6.776	26.923 ± 3.968
Elite rank group	23	30.217 ± 6.672	34.174 ± 6.443	27.870 ± 4.260
*F*-value		3.793	1.179	2.521
*p*-value		0.015	0.327	0.068

### 3.4 U-shaped curvilinear predictive effects of MOBA eSports participation duration on psychological traits of disabled individuals

Hierarchical multiple linear regression was employed to test the curvilinear predictive effect of MOBA eSports duration on self-efficacy. In the first step, only the linear term was entered, and the overall model was not significant (*F* = 0.255, *p* = 0.615, *R*^2^ = 0.005). After adding the quadratic term in the second step, the model fit improved significantly (Δ*R*^2^ = 0.546, *F* = 33.097, *p* < 0.001, adjusted *R*^2^ = 0.534). The quadratic term (B = 4.997, *p* < 0.001) was significant, indicating a U-shaped relationship with an upward opening. The VIF value was 1.015, well below 3, indicating no multicollinearity between the linear and quadratic terms, and suggesting that the model estimates are robust and reliable (see Table 5).

**Table 5:** Hierarchical regression results for the predictive effects of MOBA eSports participation duration on psychological traits of disabled individuals.

Dependent variable	Hierarchical	*F*	*p*	*R* ^2^	$\Delta R^{2}$	B	*p*	VIF
Self-efficacy	Mode 1	0.255	0.615	0.005	-	4.527E-5	1.000	1.000
						−0.439	0.615	
	Model 2	33.097	<0.001	0.551	0.546	−5.235	<0.001	1.015
						0.144	0.810	
						4.997	<0.001	
Social support	Model 1	0.003	0.960	<0.001	-	−4.877E-6	1.000	1.000
						−0.044	0.960	
	Model 2	0.227	0.798	0.008	0.008	31.308	0.625	1.015
						4.711	0.975	
						−1.056	0.505	
Trait sport confidence	Model 1	0.939	0.337	0.017	-	−8.196E-6	1.000	1.000
						−0.534	0.337	
	Model 2	6.377	0.003	0.191	0.174	−1.888	<0.016	1.015
						−0.324	<0.527	
						1.802	0.001	

Hierarchical multiple linear regression was also used to examine the predictive effect of MOBA eSports participation duration on social support. Model 1, which included only the linear term, was not significant (*F* = 0.003, *p* = 0.960, R^2^ < 0.001), and the coefficient for the linear term did not reach significance. Model 2, with the quadratic term added, also remained non-significant (*F* = 0.227, *p* = 0.798, R^2^ = 0.008, ΔR^2^ = 0.008, *p* > 0.05), with neither the linear nor the quadratic coefficient reaching significance, suggesting that participation duration has no significant linear or curvilinear predictive effect on social support.

Hierarchical regression was further applied to test the curvilinear predictive effect of MOBA eSports duration on trait sport confidence. In the first step, only the linear term was entered, and the overall model was not significant (*F* = 0.939, *p* = 0.337, *R*^2^ = 0.017). After adding the quadratic term in the second step, the model improved significantly (Δ*R*^2^ = 0.174, *F* = 6.377, *p* = 0.003, adjusted *R*^2^ = 0.161). The quadratic term (B = 1.802, *p* = 0.001) was significant, indicating a U-shaped relationship with an upward opening. The VIF value was 1.015, well below 3, indicating no multicollinearity between the linear and quadratic terms, and suggesting that the model estimates are robust and reliable.

Taken together, daily MOBA eSports participation duration showed no significant linear relationship with either self-efficacy or trait sport confidence, but rather a significant U-shaped curvilinear relationship, thereby confirming the research hypothesis.

### 3.5 Multiple linear regression results

In this study, three multiple linear regression models were constructed with self-efficacy, social support, and trait sport confidence as dependent variables, respectively. MOBA eSports participation years and eSports performance (rank) were entered as independent variables, while gender, age, marital status, education attainment, disability type, and disability grade were included as control variables. As shown in Tables 6–8, the variance inflation factor (VIF) for all independent variables ranged from 1.125 to 1.969, all below the critical value of 3, indicating no multicollinearity issues. The Durbin-Watson test values were 2.481, 1.704, and 1.446, respectively, suggesting no autocorrelation in the residuals, thus confirming that the fundamental assumptions of the regression models were satisfied.

**Table 6:** Multiple linear regression analysis of MOBA eSports participation factors on self-efficacy.

Variable	Unstandardized coefficients B	Standard error	Standardized coefficients *β*	*t*-value	*p*-value	VIF
Constant	13.778	7.082	-	1.945	0.058	-
Years of eSports participation	1.414	1.027	0.179	1.376	0.175	1.125
eSports performance (rank)	2.444	0.834	0.418	2.930	0.005^**^	1.354
Gender (male = 1)	−0.371	2.703	−0.020	−0.137	0.892	1.478
Age	−0.516	1.105	−0.065	−0.467	0.642	1.294
Marital status	0.623	1.045	0.091	0.596	0.554	1.566
Education attainment	−1.032	1.543	−0.115	−0.669	0.507	1.969
Disability type	0.676	1.101	0.085	0.614	0.542	1.276
Disability grade	2.235	1.260	0.291	1.775	0.082	1.792
*R* ^2^	0.279					
Adjusted *R*^2^	0.158					
*F*-value	2.317, *p* = 0.034^*^					
D-W value	2.481					

*p < 0.05, **p < 0.01, ***p < 0.001.

**Table 7:** Multiple linear regression analysis of MOBA eSports participation factors on social support.

Variable	Unstandardized coefficients B	Standard error	Standardized coefficients *β*	*t*-value	*p*-value	VIF
Constant	37.425	6.212	-	6.024	0.000***	-
Years of eSports participation	−0.896	0.901	−0.112	−0.995	0.325	1.478
eSports performance (rank)	−0.312	0.732	−0.053	−0.427	0.671	1.294
Gender (male = 1)	2.388	2.371	0.130	1.007	0.319	1.566
Age	−1.747	0.969	−0.217	−1.803	0.078	1.969
Marital status	3.781	1.105	0.485	3.422	0.000***	1.276
Education attainment	−0.962	0.965	−0.119	−0.997	0.324	1.792
Disability type	−0.616	1.354	−0.068	−0.455	0.651	1.354
Disability grade	0.255	0.917	0.037	0.278	0.782	1.125
*R* ^2^	0.461					
Adjusted *R*^2^	0.372					
*F*-value	5.138, *p* < 0.001					
D-W value	1.704					

*p < 0.05, **p < 0.01, ***p < 0.001.

**Table 8:** Multiple linear regression analysis of MOBA eSports participation factors on trait sport confidence.

Variable	Unstandardized coefficients B	Standard error	Standardized coefficients *β*	*t*-value	*p*-value	VIF
Constant	30.681	4.707	-	6.518	0.000^***^	-
Years of esports participation	−0.325	0.683	−0.065	−0.477	0.636	1.125
Esports performance (rank)	0.867	0.554	0.232	1.563	0.125	1.354
Gender (male = 1)	−0.846	1.797	−0.073	−0.471	0.640	1.478
Age	0.254	0.734	0.505	0.346	0.731	1.294
Marital status	−0.982	0.837	−0.200	−1.173	0.247	1.792
Education attainment	−0.944	0.731	−0.186	−1.290	0.203	1.276
Disability type	−1.774	1.026	−0.310	−1.729	0.090	1.969
Disability grade	0.697	0.695	0.160	1.004	0.321	1.566
R^2^	0.218					
Adjusted R^2^	0.088					
F-value	1.673, P = 0.130					
D-W value	1.446					

*p < 0.05, **p < 0.01, ***p < 0.001.

The overall model test showed that *F* (8, 48) = 2.317, *p* = 0.034 < 0.05, indicating that the overall model was significant, with an adjusted *R*^2^ of 0.158, meaning that the variables together explained 15.8% of the variance in self-efficacy, suggesting an acceptable model fit (see Table 6). After controlling for all confounding variables, the independent variables showed differentiated effects. MOBA eSports participation years had no significant effect on self-efficacy (*β* = 0.179, *p* = 0.175). MOBA eSports performance (rank) positively and significantly predicted self-efficacy (*β* = 0.418, *p* = 0.005), representing the most influential factor in the model, indicating that better eSports performance is associated with higher levels of self-efficacy.

The overall model test results showed that *F*(8, 48) = 5.138, *p* < 0.001, indicating that the overall model was significant (see Table 7). With an adjusted *R*^2^ of 0.372, the variables included in this study explained 37.2% of the variance in social support, demonstrating good overall explanatory power. The variance inflation factor (VIF) for all independent variables ranged from 1.125 to 1.969, all below the critical value of 5, indicating no multicollinearity issues. The Durbin-Watson test value was 1.704, suggesting no autocorrelation in the residuals, thus confirming that the fundamental assumptions of the regression model were satisfied. After controlling for all demographic and individual characteristic variables, the results showed that marital status positively and significantly predicted social support (*β* = 0.485, *p* < 0.001). However, neither MOBA eSports participation years (*β* = −0.112, *p* = 0.325) nor eSports performance (rank) (*β* = −0.053, *p* = 0.671) had significant predictive effects on social support. Overall, after controlling for relevant confounding variables, neither MOBA eSports participation years nor eSports performance effectively predicted the social support levels of disabled eSports players. Instead, marital status emerged as the key factor influencing their social support.

The overall regression model for trait sport confidence was not significant: *F*(8, 48) = 1.673, *p* = 0.130, adjusted *R*^2^ = 0.088, indicating that the model lacked statistical significance overall (see Table 8). Therefore, the individual coefficients of the predictor variables are not interpretable.

The study compared the results of the three regression analyses for self-efficacy, social support, and trait sport confidence, respectively. After controlling for demographic and disability-related variables, MOBA eSports performance showed a stable and significant positive predictive effect on self-efficacy, but showed no significant predictive effect on social support. MOBA eSports participation years had no significant effects on any of the three variables. The control variables did not generally show significant effects across the three models.

## 4 Discussion

As a mainstream form of electronic sports, MOBA (Multiplayer Online Battle Arena) games have emerged as a significant digital sports medium for individuals with disabilities, owing to their online competitive format. This study focuses on disabled MOBA players under the age of 60, drawing upon Bandura’s social cognitive theory. The aim is to examine the relationship between key participatory factors in MOBA gaming and three psychological traits among disabled players, namely general self-efficacy, social support, and trait sport confidence.

### 4.1 Differences in the impact of MOBA game participation on psychological traits of individuals with disabilities

Based on the findings of this study, research hypothesis H1 was supported, indicating that participation in MOBA games exerts a positive influence on the psychological traits of individuals with disabilities. Specifically, individuals with disabilities who participated in eSports demonstrated significantly higher levels of self-efficacy, social support, and trait athletic self-confidence compared to their non-participating counterparts.

Self-efficacy, as a core psychological construct within Social Cognitive Theory (SCT), refers to an individual’s belief in their capacity to execute tasks and achieve goals in specific situations. It is profoundly shaped by enactive mastery experience, vicarious experience, verbal persuasion, and physiological and affective states (Bandura, 2004). The MOBA gaming context aptly illustrates these four efficacy pathways, a notion corroborated by a growing body of eSports psychology research. First, in terms of enactive mastery experience, rank advancement and match victories constitute a stepped and visualized positive feedback loop. For individuals with disabilities, physical impairments often hinder the sustained attainment of achievement experiences in real-life settings, traditional sports, or vocational contexts. The points, ranking tiers, and badge systems inherent in eSports can generate a potent compensatory achievement effect, continuously reinforcing the core belief that “one is capable of overcoming challenges” (Trotter et al., 2022). Concurrently, studies on adaptive eSports have highlighted the psychological compensatory role of games; for instance, audio-centric games offer sensory substitution for visually impaired players, constructing a mediatized space they can “visualize,” thereby facilitating digital and social inclusion (Jiang, 2024). Second, at the level of vicarious experience, SCT underscores the pivotal role of observational learning in behavioral change (Caprara and Steca, 2025). For example, disabled players may acquire vicarious experiences by watching live-streamed matches, thereby bolstering their confidence in their own potential to attain similar accomplishments. Finally, regarding affective regulation, individual differences in confidence regarding emotional management directly influence affective states and coping strategies (Caprara and Steca, 2025). eSports possess emotion-regulation functions; after completing high-stakes matches and securing victories, players often transfer the stress-coping capacities cultivated in competitive settings to real-life challenges, thereby enhancing their psychological resilience in the face of adversity (Kuo et al., 2024).

In terms of trait athletic self-confidence, the MOBA gaming environment similarly constructs an empowerment system distinct from conventional contexts. Quantifiable metrics such as kill counts, win rates, and operational proficiency provide real-time feedback on ability fluctuations, enabling players to clearly perceive their progressive improvement. Enhancement of competitive skills and perseverance under adversity constitute the two core components of eSports-related confidence. Long-term observable skill gains tend to crystallize into stable athletic personality traits, which elucidates the phenomenon observed in this study whereby players perceived steady improvements in their operational performance alongside concomitant increases in athletic self-confidence. eSports establish a virtual competitive environment characterized by equality of capability, thereby neutralizing the competitive disadvantages imposed by physical impairments in real-world contexts and dismantling the entrenched barriers of traditional sports, which stratify competitive tiers based on innate physical attributes. Specialized research on spinal cord-injured eSports populations has further substantiated that customized assistive peripherals can fully compensate for motor limitations, enabling all participating disabled players to engage in formal matchmaking competitions, with competitive performance no longer constrained by congenital physical conditions (Tabacof et al., 2021). Prolonged and consistent experiences of fair competitive success tend to sediment into trait-like athletic self-confidence. This positive ability feedback, detached from real-world physical appraisal systems, persistently reshapes the self-perception of disabled players, fostering a stable competitive self-confidence unperturbed by physical limitations (Choi and Lee, 2022).

At the level of social support, the MOBA eSports context constructs a stable and sustained online social ecosystem for individuals with disabilities, effectively mitigating the constraints of limited offline social interaction. Disabled individuals with better psychological status and higher internet receptivity are more inclined to actively participate in MOBA gaming activities. Meanwhile, those with severe disabilities who face significant restrictions in offline activities also rely on online gaming as a primary avenue for leisure and social engagement. Given the persistent impediments to offline social participation, online games assume the dual function of maintaining existing social ties and forging new playmate connections, serving as a critical pathway for homebound individuals with severe disabilities to alleviate loneliness and sustain routine social connectedness (Nilsen et al., 2024).

Collectively, MOBA eSports participation demonstrates a significant correlational relationship with psychological traits.

### 4.2 Nonlinear relationship between average daily eSports participation duration and psychological traits

This study confirmed that the relationship between average daily eSports participation duration and both self-efficacy and trait athletic self-confidence is not a simple linear association but rather exhibits a typical U-shaped (opening upward) curvilinear pattern. A review of the existing literature reveals that prolonged gaming duration is associated with greater depletion of psychological resources and progressive declines in self-efficacy and self-confidence (Talik, 2026); conversely, moderate eSports participation can enhance positive psychological outcomes through skill improvement and social interaction (Gu et al., 2023). The nonlinear nature of this relationship implies that a single linear model would overlook the differential effects across varying duration intervals, potentially leading to distorted conclusions—a key source of the contradictory findings in prior research (Turel, 2019).

The positive U-shaped curve indicates that, prior to the inflection point, increases in daily MOBA gaming duration are accompanied by continuous decreases in self-efficacy and trait athletic self-confidence among disabled players; beyond the inflection point, both indicators steadily recover. Participants in the descending segment were primarily leisure-oriented, characterized by insufficient operational proficiency and a higher proportion of negative in-game feedback. Research on physical discomfort and psychological states among eSports players suggests that those with physical or sensory impairments are more prone to attributing operational errors and match losses to their own bodily limitations, thereby amplifying self-deprecating emotions (Alqabbani et al., 2025). Concurrently, during the moderate-duration phase, negative experiences tend to dominate, significantly diminishing positive psychological functioning—a pattern highly consistent with the early descending trajectory of the curve observed in this study (Przybylski and Weinstein, 2010). Deliberate practice theory provides a plausible explanation for the post-inflection recovery: frequent, stable, and sustained gaming participation constitutes effective deliberate practice, which systematically enhances core competencies such as mechanical execution, tactical reasoning, and situational judgment, while accumulating positive mastery experiences that reshape ability-related self-perceptions (Hao and Wu, 2006). For disabled players, this prolonged participation also entails a progressive process of adapting to assistive devices, including keyboard-mouse setups, motion-sensing controllers, and eye-tracking systems. Moreover, the gradual mastery of adaptive equipment and the incremental overcoming of physical operational constraints engender a pronounced sense of “self-transcendence,” which substantially reinforces self-identity and competitive confidence (Hassan, 2024). Furthermore, players with longer participation durations tend to have stable teammates, established coordination, and proficient technical skills; peer collaboration, post-match performance debriefing, and experience sharing consistently provide vicarious experiences and verbal persuasion, with the convergence of multiple efficacy-related inputs sustaining the upward trajectory of self-efficacy (Vella, 2022). As trait athletic self-confidence represents a stable personality disposition that transcends specific situations and timeframes, sustained positive competitive experiences gradually become internalized as enduring confidence tendencies, further elevating the relevant indicators. Nevertheless, prolonged maintenance of fixed postures during gaming may precipitate cervical and lumbar discomfort; for players with physical impairments, such somatic complaints may further constrain daily functioning (Kuo et al., 2024). High-intensity competitive titles like MOBA games also subject players to sustained sympathetic nervous system activation; excessively long daily participation depletes psychological resources over time, fostering persistent negative affect such as anxiety and irritability, thereby generating emotional drain (Lee, 2025). Particularly in the aftermath of defeats or during high-pressure matches, disabled players are more susceptible to negative psychological experiences, and excessive participation may amplify psychiatric symptoms including anxiety and depression (Nilsen et al., 2024).

Social support was found to be unaffected by average daily participation duration, which may be attributable to the fact that the social support measured in this study encompasses dimensions such as offline tangible assistance, affective bonding, and belonging support. Virtual social interaction serves merely as a supplementary form of offline support; individuals’ stable long-term social structures and substantive support from family and community members exhibit strong temporal stability and do not fluctuate with variations in online activity duration (Nilsen et al., 2024).

It is noteworthy that, in traditional sports, participation duration and training intensity typically demonstrate linear positive relationships with athletic confidence and self-efficacy. In contrast, eSports are subject to multiple confounding factors, including game version updates, real-time online competition dynamics, network conditions, and participants’ physical status, collectively giving rise to the distinctive U-shaped pattern identified in this study (Lochbaum et al., 2022).

### 4.3 Regression analysis of MOBA game participation years and game performance on psychological traits

#### 4.3.1 The effect of MOBA game participation years

The results revealed that eSports participation years had no significant predictive effect on self-efficacy, trait athletic self-confidence, or social support. Accordingly, the hypotheses concerning participation years in H2b and H2d were not supported, whereas H2c was fully corroborated.

This finding diverges from conclusions in traditional sports and certain professional eSports studies, which generally equate participation years among professional athletes and professional eSports players with cumulative high-intensity effective training duration, thereby closely binding years of involvement with practice quality (Papp, 2025). However, the present sample comprised amateur disabled MOBA players, whose participation was markedly intermittent. Constrained by fluctuating health conditions and daily life stressors, many participants had engaged in eSports for several years yet maintained low average daily duration, predominantly participating in casual matchmaking and bot games, with minimal involvement in high-intensity ranked or competitive play. Against this backdrop, mere temporal accumulation proved insufficient to exert the expected effects. Bandura’s self-efficacy theory emphasizes that state self-efficacy primarily relies on recent mastery experiences and current affective states, whereas the influence of distal experiences rapidly decays as contexts, skills, and environments shift. Given the rapid iteration cycles of the eSports industry, operational paradigms and game understandings from years ago gradually become obsolete; compounded by the inherent volatility in the physical conditions of disabled players, remote competitive successes fail to exert sustained influence on current competence perceptions (Kaczmarek, 2025). As for social support, the supportive resources generated through online virtual interaction are contextually confined to the digital sphere and lack permeability into the offline support systems of family and community. Thus, no transmission pathway exists for online participation behaviors to spill over into offline support, rendering neither participation duration nor participation years predictive (Chen, 2022).

In sum, this study rectifies the oversimplified public perception that “longer gaming experience begets more mature competitive psychology,” while providing empirical evidence for guiding e-sports engagement behaviors among disabled populations.

#### 4.3.2 The effect of MOBA game performance

MOBA game performance demonstrated a clear divergence in its prediction of the two psychological indicators: it significantly and positively predicted self-efficacy, whereas it exhibited only a weak positive trend toward trait athletic self-confidence that did not reach statistical significance. This result intuitively reflects the fundamental difference in responsiveness to external stimuli between state-like versus trait-like psychological variables, resonating with existing findings in sport and e-sports psychology.

Specifically, MOBA game performance indicators—including rank, win rate, and in-game performance—objectively reflect players’ comprehensive competitive capabilities. Competitive outcomes and match results constitute primary determinants of game-related self-efficacy; superior competitive performance effectively reduces competitive anxiety and reinforces operational confidence. These two factors form a bidirectional reinforcing loop, wherein strong performance enhances self-efficacy, and elevated self-efficacy in turn optimizes in-the-moment execution. For disabled players, the process of overcoming physical barriers to achieve desirable outcomes engenders a heightened sense of accomplishment distinct from that of nondisabled players, further amplifying the positive psychological effects of performance (Ye et al., 2022).

Drawing upon Bronfenbrenner’s ecological systems theory, social support can be stratified into three tiers: the micro-system of family caregiving, the meso-system of community care, and the macro-system of online e-sports communities. Empirical evidence confirms that e-sports competitive performance operates exclusively within the online community tier and does not permeate the predictive models of family or community offline systems; online competitive achievements do not alter the level of family caregiving provision or real-world community interpersonal care (Yang et al., 2026).

## 5 Conclusion

This study examined the associations between MOBA eSports participation and self-efficacy, social support, and trait sport confidence among individuals with disabilities, and tested the predictive roles of participation-related characteristics.

At the between-group level, the eSports participation group scored significantly higher than the non-participation group on all three psychological indicators. This indicates a stable positive association between eSports participation and favorable psychological traits in this population. At the participation-characteristic level, average daily gaming duration showed a significant U-shaped relationship with self-efficacy and trait sport confidence. Daily participation duration was not significantly associated with social support. eSports performance (rank) significantly and positively predicted self-efficacy. In contrast, years of participation predicted none of the three indicators. This suggests that recent perceptible achievements, rather than mere time accumulation, are the primary driver of capability beliefs.

In summary, MOBA eSports participation is positively associated with favorable psychological traits in individuals with disabilities. However, the U-shaped relationship with daily gaming duration highlights the importance of moderation. Competitive performance emerges as a key predictor of self-efficacy, whereas social support appears to depend primarily on offline intimate relationships. eSports may serve as a digital medium for psychological empowerment in this population.

## 6 Limitations and future research directions

Although this study provides valuable preliminary evidence for understanding the psychological impact of eSports participation on individuals with disabilities, several limitations warrant careful consideration when interpreting the findings.

First, concerns regarding sample representativeness and generalizability. This study employed a convenience sampling method and ultimately included 114 Chinese participants. Among them, the e-sports group comprised only 57 participants, a relatively modest sample size that is particularly concerning given the number of predictors included in the regression models, which may limit the statistical power to detect true effects. Additionally, the sample was confined to a specific gaming environment and cultural context. Consequently, caution should be exercised when extending these conclusions to other eSports genres—such as first-person shooter (FPS) and real-time strategy (RTS) games—or to disability populations in other countries and regions. Future research should recruit larger and more culturally and contextually diverse samples, especially for the e-sports subgroup, to enhance statistical power and strengthen the external validity of the findings.

Second, the cross-sectional design precludes causal inference. Although we observed significant associations between eSports participation and psychological outcomes, the direction of causality remains ambiguous—it cannot be ruled out that individuals with higher baseline self-efficacy may be more inclined to engage in eSports activities. Longitudinal studies that follow participants over time are needed to elucidate the underlying causal mechanisms.

Third, the singular focus on one game genre limits generalizations about eSports as a whole. This study concentrated exclusively on a single MOBA title (Honor of Kings), a choice that, while facilitating internal consistency, raises questions about the transferability of our results to other game types that impose different demands on reaction time, teamwork, or communication modalities. Accordingly, unless further evidence emerges, any assertions regarding “eSports” in this paper should be understood as specifically referencing MOBA games. Future investigations should examine whether analogous effects can be replicated across diverse gaming genres.

Fourth, mediating mechanisms were not explored. This study did not examine the pathways through which eSports participation may influence psychological outcomes. Factors such as team cohesion, coach support, or specific in-game achievements may play pivotal mediating roles. Subsequent research employing mediation models is warranted to disentangle these underlying processes.

Fifth, key boundary conditions and moderating variables were insufficiently examined. Our sample comprised only individuals with hearing and speech impairments, excluding those with intellectual disabilities, and we did not collect data on the use of assistive technologies such as eye-tracking devices. Moreover, we did not distinguish between solo and team-based gameplay, nor did we assess within-team social support and cohesion—all of which may moderate the observed effects.

Sixth, inadequate attention was paid to the clinical risks associated with excessive gaming. This study characterized the subgroup with daily gaming sessions exceeding 8 h as “exhibiting adaptive occupational traits,” yet failed to adequately address the potential pathological ramifications, including gaming disorder, compulsive gaming behaviors, and psychological dysfunction resulting from excessive screen exposure. This framing—which construes extreme gaming duration as indicative of “high adaptation” while overlooking its clinical hazards—presents a logical inconsistency from a public health perspective. Although this study emphasizes the positive psychological effects of moderate eSports engagement among individuals with disabilities, it does not concurrently examine the dose-dependent negative consequences of excessive play. Future research should adopt a more balanced approach that comprehensively evaluates both the benefits and drawbacks of eSports participation, with particular attention to the clinical risks of chronic excessive gaming, thereby providing more nuanced and evidence-based guidance for the healthy integration of eSports into the lives of disability populations.

Regarding demographic variables and disability-related characteristics, our findings indicate that marital status, as an indicator of offline intimate relationships, significantly influenced psychological outcomes—married individuals benefited from sustained daily care, emotional companionship, and material support from their partners, constituting a vital source of social support consistent with the fundamental characteristics of social support in disability populations. Gender, age, and disability grade did not exhibit pervasive effects, suggesting that, within the context of eSports participation, competitive behaviors and in-game experiences may partially attenuate the influence of demographic traits and physical impairments per se on psychological attributes. Additionally, disability type demonstrated a marginally significant trend in relation to trait sport confidence, hinting at subtle differences in competitive mindset across disability types that merit further investigation through stratified analyses.

In summary, this study offers preliminary support for the positive role of eSports in promoting psychological well-being among individuals with disabilities; however, the potential risks and boundary conditions of these effects necessitate more systematic and rigorous exploration. Future research should prioritize improvements in sample diversity, longitudinal design, mechanistic precision, and comprehensive risk assessment to yield more robust conclusions and practically actionable recommendations.

## Data Availability

The raw data supporting the conclusions of this article will be made available by the authors, without undue reservation.
